# Acute liver failure in Japan: definition, classification, and prediction of the outcome

**DOI:** 10.1007/s00535-012-0624-x

**Published:** 2012-07-24

**Authors:** Kayoko Sugawara, Nobuaki Nakayama, Satoshi Mochida

**Affiliations:** Department of Gastroenterology and Hepatology, Faculty of Medicine, Saitama Medical University, 38 Morohongo, Moroyama-cho, Iruma-gun, Saitama 350-0495 Japan

**Keywords:** Acute liver failure, Fulminant hepatitis, Late onset hepatic failure, Hepatic encephalopathy, Liver transplantation

## Abstract

Acute liver failure is a clinical syndrome characterized by hepatic encephalopathy and a bleeding tendency due to severe impairment of liver function caused by massive or submassive liver necrosis. Viral hepatitis is the most important and frequent cause of acute liver failure in Japan. The diagnostic criteria for fulminant hepatitis, including that caused by viral infections, autoimmune hepatitis, and drug allergy induced-liver damage, were first established in 1981. Considering the discrepancies between the definition of fulminant hepatitis in Japan and the definitions of acute liver failure in the United States and Europe, the Intractable Hepato-Biliary Disease Study Group established the diagnostic criteria for “acute liver failure” for Japan in 2011, and performed a nationwide survey of patients seen in 2010 to clarify the demographic and clinical features and outcomes of these patients. According to the survey, the survival rates of patients receiving medical treatment alone were low, especially in those with hepatic encephalopathy, despite artificial liver support, consisting of plasma exchange and hemodiafiltration, being provided to almost all patients in Japan. Thus, liver transplantation is inevitable to rescue most patients with hepatic encephalopathy. The indications for liver transplantation had, until recently, been determined according to the guideline published by the Acute Liver Failure Study Group in 1996. Recently, however, the Intractable Hepato-Biliary Disease Study Group established a scoring system to predict the outcomes of acute liver failure patients. Algorithms for outcome prediction have also been developed based on data-mining analyses. These novel guidelines need further evaluation to determine their usefulness.

## Introduction

Liver failure is a clinical syndrome characterized by jaundice, ascites, hepatic encephalopathy, and a bleeding tendency due to impairment of liver function; the syndrome can be caused by conditions such as viral hepatitis, autoimmune hepatitis, drug-induced liver injuries, metabolic diseases, and circulatory disturbances. Liver failure has been classified into 2 types, depending on the clinical course; namely, acute liver failure and chronic liver failure. In general, acute liver failure is diagnosed in patients in whom severe liver function impairment, as judged from the clinical symptoms, laboratory data, and imaging examinations, develops within 24 or 26 weeks (6 months) following the onset of liver injury in a preexisting normal liver, while chronic liver failure is diagnosed in patients with persistent liver inflammation and injuries who show liver function impairment later than 6 months after the onset of the liver symptoms.

Worldwide, the representative disease entity associated with chronic liver failure is liver cirrhosis due to persistent hepatitis virus infection, autoimmune hepatitis, or hepatitis of indeterminate etiology. However, the demographic and clinical features of acute liver failure differ between Japan and Europe or the United States. Hepatitis viral infection is the most important and common cause of acute liver failure in Japan [1], while drug-induced liver injury, including acetaminophen intoxication, is the major cause of acute liver failure in Europe and the United States [2]. Thus, acute liver failure is typically associated with fulminant viral hepatitis in Japan, and the diagnostic criteria for “fulminant hepatitis”, which are different from those for “acute liver failure” in Europe and the United States, were first established at the Inuyama Symposium in 1981 [3].

The therapeutic strategies for acute liver failure also differ between Japan and Europe and/or the United States. Cadaveric transplantation is considered as the first-line therapy for acute liver failure in Europe and the United States [2], while in Japan, artificial liver support, consisting of plasma exchange and hemodiafiltration, is usually provided first to patients with fulminant hepatitis [1], and liver transplantation, usually from living donors [4–6], is only scheduled when no clinical improvement is achieved with this conventional medical treatment [1]. Thus, the criteria for scheduling liver transplantation for patients with acute liver failure and/or fulminant hepatitis also differ between Japan and other countries.

In the present article, we discuss in detail the differences between Japan and Europe and/or the United States in the definitions, classifications, and diagnostic criteria for the disease subtypes of acute liver failure and/or fulminant hepatitis, and also the indications for liver transplantation in these patients. Also, we describe the clinical features of patients with acute liver failure in Japan, which were analyzed through a nationwide survey conducted using the diagnostic criteria revised in 2011 in reference to those in Europe and the United States.

## Diagnostic criteria for acute liver failure in Europe, the United States, and Asian countries other than Japan

Epidemic hepatitis was a worldwide health problem in the early twentieth century, and it was already known, even in the 1920s, that differences existed in the clinical course even among patients with the fatal form of the disease, and that patients could be divided into those with fulminant, acute, or chronic forms [7]. Although most patients with fatal disease exhibited a subacute clinical course, with the patients dying between 4 and 6 weeks after the onset of the hepatitis symptoms, a patient showing a more rapid course was first reported in the great Swedish epidemic in 1927 [8]. This was the first case report of fulminant-type epidemic hepatitis although it was thought that such patients were seldom encountered elsewhere in the world. However, the concept of epidemic hepatitis changed with the occurrence of World War II; epidemic hepatitis changed to a hepatitis pandemic during World War II, with large outbreaks occurring in many parts of the world, especially in armies. Lucke and Mallory [7] reported on the outbreak of epidemic hepatitis in the United States Army between August 1943 and April 1945, in which 104 of 196 patients (53 %) had a fatal course and died within 10 days of the onset of the hepatitis symptoms. Lucke and Mallory named this form of hepatitis, which was probably caused by hepatitis A virus (HAV) or hepatitis E virus (HEV) infection, the “fulminant form” of hepatitis.

By the end of World War II, the hepatitis pandemic had not spread to any extent in Europe or the United States, and the “fulminant form of hepatitis” was recognized as an infrequent, but intractable, disease in Western countries. Also, advances in the field of virology in the 1970s enabled hepatologists to evaluate patients with fulminant hepatitis for serum markers of HAV and hepatitis B virus (HBV) infection, and these two types of hepatitis viruses were found to account for a large proportion of the patients [9–11]. On the other hand, Trey et al. [12] reported that drugs such as halothane may also cause acute liver disease, similar in clinical course to fulminant hepatitis. They evaluated the etiology in 150 patients enrolled from 73 centers, and revealed that the cause of the hepatitis was presumed viral hepatitis in 70 patients (46.7 %) (including serum hepatitis and epidemic hepatitis in 24 and 46 patients, respectively), and drug-induced liver disease in 48 patients (32.0 %) (including 36 with halothane exposure as the culprit) [12]. These observations prompted hepatologists to use the nomenclature of “fulminant hepatic failure” instead of “fulminant hepatitis” for patients presenting with acute onset of massive liver necrosis. Then, in 1970, Trey and Davidson [13] proposed the now well-known diagnostic criteria for fulminant hepatic failure; they defined the condition as a clinical syndrome characterized by massive liver necrosis associated with severe impairment of hepatic function, manifesting as progressive jaundice, hepatic coma, and liver atrophy developing within 8 weeks of the onset of the first symptoms of the disease in individuals with no previous history of hepatic disease. Moreover, in 1986, Gimson et al. [14] suggested that patients showing hepatic encephalopathy as well as other evidence of hepatic decompensation developing more than 8 weeks but less than 24 weeks of the onset of the first symptoms be labeled as having late-onset hepatic failure (LOHF), a clinical syndrome related to fulminant hepatic failure.

In contrast, in France, Bernuau et al. [15] proposed the nomenclature of “fulminant and subfulminant liver failure” for patients with rapidly progressive hepatic failure; patients developing hepatic encephalopathy less than 2 weeks after the onset of jaundice were diagnosed as having fulminant liver failure, while those with hepatic encephalopathy developing between 2 and 12 weeks after the onset of jaundice were labeled as having subfulminant liver failure. Also, the terminology of “subacute liver failure” was introduced in India in 1982 for patients showing progressive jaundice with ascites of 8 weeks’ duration, with otherwise typical features of acute viral hepatitis; the presence of hepatic encephalopathy was not a necessary criterion for the diagnosis of this condition [16]. While intense debate still continues on the definition and classification of patients showing rapid progression of hepatic failure, all hepatologists around the world, including those in Japan [17], agree that patients showing the most rapid onset of hepatic encephalopathy have the best chance of recovery with conventional medical treatment. Although geographic heterogeneity was found in the etiology and clinical features of acute liver diseases, standardization of the nomenclature and diagnostic criteria for patients showing rapidly progressive hepatic failure is required for reliable comparisons of the efficacies of various treatments and of the resultant outcomes among different countries.

Thus, in 1993, O’Grady et al. [18] redefined such syndromes as “acute liver failure” prefixed with “hyper” and “sub” to describe 2 cohorts at opposite ends of the clinical spectrum, based on the observation of a large series of patients treated at King’s College Hospital, London, between 1972 and 1985. According to this proposed classification, patients with hepatic encephalopathy developing within 7 days of the onset of jaundice are diagnosed as having “hyperacute liver failure”, which is often caused by acetaminophen intoxication [18]. In contrast, patients showing hepatic encephalopathy between 8 and 28 days and those with encephalopathy developing later than 28 days after the first onset of symptoms were diagnosed as having “acute liver failure” and “subacute liver failure”, respectively, with HAV or HBV infection and drug-induced liver damage being more frequently seen in the former cohort, and liver disease of indeterminate etiology being seen more frequently in the latter cohort [18]. It should be noted that patients with pre-existing symptomless chronic liver diseases were included in the disease entity of “acute liver failure” by O’Grady et al. [18], and in that of “fulminant liver failure” by Bernuau et al. [15], while such patients were excluded from the entity of “fulminant hepatic failure” by Trey and Davidson [13].

Criticisms were raised regarding the nomenclature and definition of “acute liver failure” proposed by the King’ College Hospital group, especially in France [19] and India [20]. Thus, the subcommittee of the International Association for the Study of the Liver (IASL) published revised recommendations on the nomenclature of acute and subacute hepatic failure in 1999 [21]. In this recommendation, two distinct disease entities, but not subgroups of a syndrome, were established; namely, “acute hepatic failure” and “subacute hepatic failure”. Patients without pre-existing liver disease developing hepatic encephalopathy within 4 weeks of the onset of the disease symptoms are diagnosed as having acute hepatic failure. Acute hepatic failure is a potentially reversible liver disease and is classified into hyperacute and fulminant forms, defined by the development of hepatic encephalopathy less than 10 days and between 10 and 30 days, respectively, after the first onset of the disease symptoms. In contrast, patients developing hepatic encephalopathy between the 5th and 24th weeks after the first onset of symptoms are diagnosed as having subacute hepatic failure.

Consequently, until the beginning of the twenty-first century, hepatitis showing rapid progression was referred to by various names, including fulminant hepatitis [7], fulminant hepatic failure [13], fulminant liver failure [15], acute liver failure [18], and acute hepatic failure [21] (Table 1). However, “acute liver failure” came to be used predominantly as the most suitable umbrella term, because it can be assumed to include all of the other disease entities [22]. Thus, the Practice Guideline Committee of the American Association for the Study of Liver Diseases (AASLD) published a position paper for the management of “acute liver failure” in 2005 [2]. Acute liver failure is defined as “liver diseases characterized by the development of hepatic encephalopathy and coagulation abnormalities, usually characterized by an international normalized ratio (INR) of 1.5 or more, in patients without preexisting cirrhosis, and an illness of less than 26 weeks duration”. In this position paper, subgroups classified according to the interval between the onset of hepatic encephalopathy and the first onset of the disease symptoms; namely, the hyperacute, acute, and subacute types, were shown to be not helpful to predict the outcomes of the patients [2]. Despite the publication of this position paper by the AASLD, differences in the definitions are still seen in recent articles regarding acute liver failure [23], and these differences hamper the conduct of reliable meta-analyses.

**Table 1:** Definition and classification of acute liver failure and related diseases

Year	Nomenclature (classification)	Parameters			Author	Affiliation or country	Reference
		Development of hepatic encephalopathy	PT	Pre-LD			
1930	Acute yellow liver dystrophy	ND	–	–	Bergstrand	Germany	[8]
1946	Fulminant form of epidemic hepatitis	ND	–	–	Lucke and Mallory	USA	[7]
1970	Fulminant hepatic failure	Within 8 weeks after disease symptoms onset	–	Absent	Trey and Davidson	USA	[13]
1981	Fulminant hepatitis (acute & subacute)	Within 8 weeks after disease symptoms onset (within 10 days and between 11 and 56 days, respectively)	≤40 %	Absent	Takahashi et al.	Japan	[3]
1982	Subacute hepatitis	Not necessarily present	–	–	Tandon et al.	India	[16]
1986	Late onset hepatic failure	Between 8 and 24 or 26 weeks after disease symptoms onset	–	–	Gimson et al.	England	[14]
1986	Fulminant liver failure and subfulminant liver failure	Less than 2 weeks and between 2 and 12 weeks, respectively, after jaundice onset	–	Absent or present	Bernuau et al.	France	[15]
1993	Acute liver failure (hyperacute, acute and subacute)	Within 7 days, between 8 and 28 days, and later than 28 days, respectively, after jaundice onset	–	Absent or present	O’Grady et al.	England	[18]
1999	Acute hepatic failure (hyper-acute and fulminant) and subacute hepatic failure	Within 4 weeks (less than 10 days and between 10 and 30 days) and between 5th and 24th weeks, respectively, after disease symptoms onset	–	–	Tandon et al.	IASL	[21]
2005	Acute liver failure	Preexisting illness of less than 26 weeks’ duration	Usually INR ≥1.5	Absent	Polson and Lee	AASLD	[2]
2011	Acute liver failure [without or with coma (acute and subacute)]	Without or with encephalopathy within 8 weeks of disease symptoms onset (within 10 days and between 11 and 56 days, respectively)	≤40 % or INR ≥1.5	Absent	Mochida et al.	Japan	[30]

*PT* prothrombin time, *Pre-LD* preexisting symptomless liver diseases, *ND* not described, *IASL* International Association for the Study of the Liver, *AASLD* American Association for the Study of Liver Diseases

## Definition, classification, and diagnostic criteria for fulminant hepatitis and acute liver failure in Japan

The definition and classification of fulminant hepatitis, the representative disease entity associated with acute liver failure in Japan, were established at the Inuyama Symposium in 1981 [3]. According to the Inuyama Symposium criteria, patients with hepatitis were diagnosed as having fulminant hepatitis when they developed grade II or more severe hepatic encephalopathy due to severe liver damage, as represented by prothrombin time values of ≤40 % of the standardized value, within 8 weeks of the onset of the hepatitis symptoms. Fulminant hepatitis was further classified into 2 clinical types; that is, the acute and subacute types, on the basis of the hepatic encephalopathy developing within 10 days or between 11 and 56 days, respectively, after the onset of the hepatitis symptoms. Fulminant hepatitis in Japan is defined as histological evidence of hepatic inflammation, characterized by lymphocytic infiltration of the liver, associated with acute liver failure. Thus, the etiology of fulminant hepatitis comprises viral infections, including HBV carriers, autoimmune hepatitis, drug allergy-induced liver injuries, and hepatitis of indeterminate etiology.

The Intractable Liver Diseases Study Group of Japan, supported by the Ministry of Health, Labour and Welfare, last revised the diagnostic criteria for “fulminant hepatitis” in 2002 (Table 2) [1]. In this revision, 5 items clarifying the inclusion and exclusion criteria for the diagnosis of fulminant hepatitis were added as a footnote. We clarified that patients with preexisting chronic liver diseases, such as those with alcoholic hepatitis, were excluded from the disease entity of fulminant hepatitis, whereas asymptomatic HBV carriers developing acute exacerbation of hepatitis were included as cases of fulminant hepatitis. Also, the significance of histological evidence of liver inflammation was emphasized, so that liver failure caused by drug or chemical intoxication, circulatory disturbances, acute fatty liver of pregnancy, Reye’s syndrome, or Wilson disease were excluded from the diagnosis of fulminant hepatitis. Moreover, the definitions of subtypes of fulminant hepatitis were clarified; hepatitis patients with no or grade I encephalopathy, but showing prothrombin time values of ≤40 % of the standardized value were diagnosed as having “severe type of acute hepatitis”, while those with grade II or more severe hepatic encephalopathy developing between 8 and 24 weeks after the disease symptoms onset, with prothrombin time values of ≤40 % of the standardized value were diagnosed as having LOHF. Thus, the disease entity of LOHF in Japan differed from that in Europe and the United States [14], because patients with no histological evidence of hepatitis and/or prothrombin time values of >40 % of the standardized value were excluded from the diagnosis of LOHF in Japan, even if they had grade II or more severe hepatic encephalopathy.

**Table 2:** Diagnostic criteria for fulminant hepatitis in Japan established by the Intractable Liver Diseases Study Group of Japan, supported by the Ministry of Health, Welfare and Labour (2003); from reference [1]

Fulminant hepatitis is defined as hepatitis with hepatic encephalopathy of grade II or more that develops in the patients within 8 weeks of the onset of disease symptoms, associated with severe derangement of liver function, including prothrombin time values of less than 40 % of the standardized value. Fulminant hepatitis is classified into 2 subtypes; the acute type and the subacute type, according to whether the encephalopathy occurs within 10 days and later than 11 days, respectively, after the onset of the symptoms.
Note 1: Patients with chronic liver diseases are excluded from the disease entity of fulminant hepatitis, but asymptomatic hepatitis B virus (HBV) carriers developing acute exacerbation are included as cases of fulminant hepatitis.
Note 2: Acute liver failure with no histological evidence of liver inflammation, such as that caused by drug or chemical intoxication, circulatory disturbance, acute fatty liver of pregnancy, or Reye’s syndrome is excluded from the disease entity of fulminant hepatitis.
Note 3: The grading of hepatic encephalopathy is based on the criteria presented at the Inuyama Symposium in 1972.
Note 4: The etiology of fulminant hepatitis is based on the criteria established by the Intractable Liver Diseases Study Group of Japan in 2002.
Note 5: Patients with no or grade I encephalopathy, but showing prothrombin time values of less than 40 % of the standardized value are diagnosed as having acute hepatitis, severe type. Patients in whom the encephalopathy develops between 8 and 24 weeks after the disease onset, with prothrombin time values of less than 40 % of the standardized value are diagnosed as having late-onset hepatic failure (LOHF). Both are diseases related to fulminant hepatitis, but are regarded differently from fulminant hepatitis.

In the revision conducted in 2002, however, the definition and concept of fulminant hepatitis were not modified, because the diagnostic criteria established at the Inuyama Symposium were useful to characterize the clinical features of patients with acute liver failure in Japan. According to the nationwide survey of fulminant hepatitis and LOHF conducted by the Intractable Hepato-Biliary Diseases Study Group in Japan (formally the Intractable Liver Diseases Study Group of Japan), the clinical features of patients differed markedly when the disease types were defined based on the diagnostic criteria established in 1981 or based on the revised criteria established in 2002. In this survey, 1,094 patients with fulminant hepatitis, consisting of 543 with the acute type and 551 with the subacute type of fulminant hepatitis were included, and 92 patients with LOHF seen between 1998 and 2009 were enrolled [1, 24–29]. In regard to the etiology of hepatitis, viral infection accounted for 67.4 and 30.9 % of the patients with the acute and subacute types of fulminant hepatitis, respectively, and for 10.9 % of the patients with LOHF. In most patients with fulminant hepatitis caused by viral infection, irrespective of the disease type, the causative agent was HBV; transient HBV infection was more frequent in patients with the acute type (39.2 %) as compared to the subacute type (10.0 %) of fulminant hepatitis, while the frequency of HBV carriers was greater in patients with the subacute type (17.9 %) as compared to the acute type (7.2 %) of fulminant hepatitis. Autoimmune hepatitis was found in 1.8, 12.2, and 19.6 % of patients with the acute and subacute types of fulminant hepatitis and LOHF, respectively. Drug allergy-induced liver injury was seen in 9.0, 13.1, and 18.7 % of patients with the acute and subacute types of fulminant hepatitis and LOHF, respectively. It is noteworthy that the etiology was indeterminate in 19.0, 40.8, and 40.2 % of patients with the acute and subacute types of fulminant hepatitis and LOHF, respectively. The outcomes of the patients also differed between these disease types. The survival rates of patients receiving medical treatment without liver transplantation were 53.7, 24.4, and 11.5 %, respectively, in patients with the acute and subacute types of fulminant hepatitis and LOHF seen between 1998 and 2003 [1], while these rates were 48.7, 24.2, and 13.0 %, respectively, in the corresponding categories of patients seen between 2004 and 2009 [24–29].

Although the diagnostic criteria for fulminant hepatitis in Japan [1, 3] merit consideration in clinical practice for the diagnosis of acute liver failure patients, they do need to be revised to fit with the criteria for acute liver failure adopted in Europe and the United States [2]. Thus, in 2006, the Intractable Hepato-Biliary Diseases Study Group in Japan constituted a task force to establish novel diagnostic criteria for “acute liver failure”, which includes the disease entity “fulminant hepatitis”. To establish such criteria for defining “acute liver failure” in Japan, two types of nationwide surveys were performed [30]; a survey of the commercial kits used for the measurement of prothrombin time at institutions to which hepatology specialists were affiliated, and a survey of acute liver failure patients who were excluded from the disease entities of fulminant hepatitis and LOHF. Consequently, “acute liver failure” in Japan (Table 3) came to be defined as an acute liver disease associated with prolongation of the prothrombin time, with an INR of 1.5 or more. To confirm the correspondence between the present criteria (Table 3) and previous criteria (Table 2), “prothrombin time values of≤40 % of the standardized value” was also employed as a cutoff to define patients with acute liver failure. Patients without hepatic encephalopathy were also included in the disease entity of acute liver failure, if they showed an INR of 1.5 or more. Thus, acute liver failure patients are classified into those with and without hepatic coma, and acute liver failure with hepatic coma is further subdivided into 2 disease types; namely, the “acute type” and the “subacute type,” according to the interval from the onset of symptoms to the development of hepatic encephalopathy, similar to the case for fulminant hepatitis [1, 3].

**Table 3:** Diagnostic criteria for acute liver failure in Japan (2011); from reference [30]

Patients showing prothrombin time values of 40 % or less of the standardized value, or international normalized ratios (INRs) of 1.5 or more due to severe liver damage within 8 weeks of the onset of disease symptoms are diagnosed as having “acute liver failure”, where the liver function prior to the current onset of liver damage is estimated to have been normal based on blood laboratory data and imaging examinations. “Acute liver failure” is classified into “acute liver failure without hepatic coma” and “acute liver failure with hepatic coma”; no or grade I hepatic encephalopathy is present in the former type, while grade II or more severe hepatic encephalopathy is found in the latter type. “Acute liver failure with hepatic coma” is further subclassified into 2 disease types; the “acute type” and “subacute type”, with grade II or more severe hepatic encephalopathy developing within 10 days or between 11 and 56 days after the onset of disease symptoms, respectively, in the two types.
Note 1: Hepatitis B virus (HBV) carriers and autoimmune hepatitis patients showing acute exacerbation of hepatitis in the normal liver are included under the disease entity of “acute liver failure”. In the case of indeterminate previous liver function, the patients who are HBV carriers and those with autoimmune hepatitis are diagnosed as having “acute liver failure” when no liver function impairment preceding the exacerbation of the liver injury can be confirmed.
Note 2: In general, alcoholic hepatitis develops in patients with chronic liver diseases caused by habitual alcohol consumption. Thus, patients with alcoholic hepatitis are excluded from the disease entity of “acute liver failure”. However, patients with fatty liver caused by alcohol intake and those with metabolic syndrome, including obesity, are diagnosed as having “acute liver failure” if etiologies other than habitual alcohol consumption are responsible for the acute injury in the liver, in the absence of prior impairment of liver function.
Note 3: Patients without histological evidence of hepatitis, such as inflammatory lymphocytic infiltration, are included under the disease entity of “acute liver failure”. Thus, patients with liver damage caused by drug toxicity, circulatory disturbance, or metabolic disease and acute fatty liver of pregnancy are diagnosed as having “acute liver failure”, while they are excluded from the disease entity of “fulminant hepatitis”. In contrast, patients with liver injury caused by viral infection, autoimmune hepatitis, and drug allergy-induced hepatitis are included under the disease entities of “fulminant hepatitis” and “acute liver failure”.
Note 4: The severity of hepatic encephalopathy is diagnosed according to the classification presented at the Inuyama Symposium in 1972 (Table 4). Also, hepatic encephalopathy developing in pediatric patients and infants is classified according to the criteria proposed by the 5th Workshop on Pediatric Liver Diseases in 1988 (Table 5).
Note 5: The etiology of “acute liver failure” is classified according to the criteria proposed by the Intractable Liver Diseases Study Group of Japan in 2002, with some modifications (Table 6).
Note 6: Patients showing prothrombin time values of less than 40 % of the standardized value or INRs of 1.5 or more and grade II or more severe hepatic coma between 8 and 24 weeks of the onset of disease symptoms are diagnosed as having late-onset hepatic failure (LOHF), as a disease related to “acute liver failure”.

Similar to the entity of acute liver failure in Europe and the United States [2], in Japan patients without histological evidence of inflammation in the liver, such as those with the disease caused by drug toxicity, circulatory disturbances, or metabolic diseases are also included in the disease entity of acute liver failure. In contrast, patients showing impaired liver function due to underlying chronic liver diseases before the worsening of the liver damage are excluded from the disease entity of acute liver failure. Thus, alcoholic liver disease patients are excluded from this entity, because they show clinical features consistent with acute-on-chronic liver disease. However, patients with underlying chronic liver diseases such as fatty liver and autoimmune hepatitis are included in the disease entity of acute liver failure, when the liver function impairment is retrospectively estimated to be minimal or absent prior to the current exacerbation of the liver damage. Also, the criteria for classification of hepatic encephalopathy and etiology of hepatitis have been added as footnotes to the present criteria (Tables 4, 5, 6). In addition, patients with LOHF are defined as those showing prothrombin time values of ≤40 % of the standardized value or INRs of 1.5 or more and grade II or more severe hepatic coma between 8 and 24 weeks of the onset of the disease symptoms, and those without histological evidence of hepatitis are also included in the disease entity of LOHF, similar to the case of acute liver failure. On the other hand, the disease entity of “acute hepatitis severe type” was excluded from the footnote of the present criteria, because patients classified under such a disease entity can also be diagnosed as having “acute liver failure without hepatic encephalopathy”.

**Table 4:** Classification of hepatic encephalopathy in adult patients according to the grade of hepatic coma proposed by the Inuyama Symposium in 1972; from reference [30]

Grade of coma	Psychiatric disorders	Reference items
I	Inversion of sleep pattern Euphoria and/or occasional depression Negligent attitude with shortened attention span	Recognized retrospectively in most cases
II	Disorientation of time or place and confusion Inappropriate behaviors, such as throwing away money or discarding items of value Occasional somnolent tendency; able to open eyes and respond appropriately to questions Makes impolite remarks, but follows doctors’ instructions	Excitation state and, urinary and fecal incontinence are absent, but flapping tremor is found on physical examination
III	State of excitation and/or delirium, showing defiant behavior Somnolent tendency; sleeping most of the time Opens eyes in response to stimulation, but cannot follow the instructions of doctors, except for simple orders	Flapping tremor is observed, and the extent of disorientation is severe
IV	Coma; complete loss of consciousness Response to painful stimuli	Brushes off doctor’s hands if touched and/or frowns in response to stimuli
V	Deep coma No response to painful stimuli

**Table 5:** Classification of hepatic encephalopathy in pediatric patients and infants according to the grade of hepatic coma as proposed at the 5th Workshop on Pediatric Liver Diseases in 1988; from reference [30]

Grade of coma	Pediatric patients	Infants
I	Low-spirited from before (seems lethargic compared with previous physical activity level)	Does not laugh aloud
II	Obedient attitude with somnolent tendency Disorientation of time or place	Does not laugh even when being played with Cannot maintain eye contact with the mother (more than 3 months after birth)
III	Opens eyes in response to loud voice	
IV	Does not wake up in response to painful stimuli, but frowns and/or brushes off the item producing the stimulus with his/her hands	
V	No response to painful stimuli

**Table 6:** Classification of etiologies of acute liver failure modified from the criteria proposed by the Intractable Liver Diseases Study Group of Japan in 2002; from reference [30]

I.	Viral infection
	1 Hepatitis A virus (HAV)
	2 Hepatitis B virus (HBV)
	(1) Transient infection
	(2) Acute exacerbation in HBV carrier
	i. Inactive carrier, without drug exposure
	ii. Reactivation in inactive carrier by immunosuppressant and/or anticancer drugs
	iii. Reactivation in transiently infected patients by immunosuppressant and/or anticancer drugs (de-novo hepatitis)^a^
	(3) Indeterminate infection patterns
	3 Hepatitis C virus (HCV)
	4 Hepatitis E virus (HEV)
	5 Other viruses
II.	Autoimmune hepatitis
III.	Drug-induced liver injuries
	1. Drug allergy-induced liver injury
	2. Drug toxicity-induced liver injury
IV.	Circulatory disturbance
V.	Infiltration of the liver by malignant cells
VI.	Metabolic diseases
VII.	Liver injuries after liver resection and transplantation
VIII.	Miscellaneous etiologies
IX.	Indeterminate etiology despite sufficient examinations
X.	Unclassified due to insufficient examinations

Patients with etiologies I, II, and III-1 are diagnosed as having “fulminant hepatitis” as well as “acute liver failure”, whereas those with etiologies III-2 and IV to VIII are diagnosed as having “acute liver failure”, but are excluded from the disease entity of “fulminant hepatitis”. Diagnostic criteria for the classification of etiology based on laboratory data should be established in the future

^a^Serum hepatitis B surface (HBs) antigen-negative patients following transient infection with HBV are classified as HBV carriers, in whom HBV reactivation can be induced by immunosuppressant and/or anticancer drugs; however, the significance of this causative etiology needs to be evaluated further

## Nationwide survey of patients with acute liver failure in Japan

**Fig. 1:**
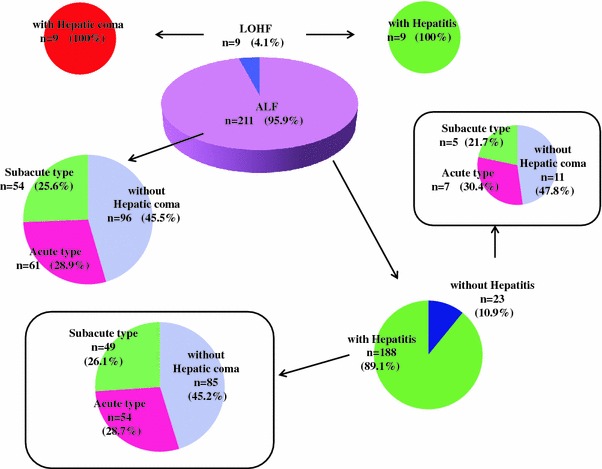
Classification of acute liver failure patients in Japan enrolled in a nationwide survey performed by the Intractable Hepato-Biliary Diseases Study Group in Japan [31]. *ALF* acute liver failure, *LOHF* late-onset hepatic failure

Recently, the Intractable Hepato-Biliary Diseases Study Group performed a nationwide survey of patients with acute liver failure seen in 2010, in whom the diagnosis was made according to the criteria published in 2011 [30]. The 220 patients, consisting of 211 patients with acute liver failure and 9 patients with LOHF, were enrolled from 742 institutions with specialists in the fields of hepatology, gastroenterology, and/or acute medicine [31]. The 211 acute liver failure patients were classified into 96 (45.5 %) without hepatic coma and 115 patients (54.5 %) with hepatic coma, with the latter group being further divided into 61 patients (28.9 %) with the acute type and 54 patients (25.6 %) with the subacute type (Fig. 1). Also, the acute liver failure patients were classified into 188 patients (89.1 %) with hepatitis and 23 patients (10.9 %) without hepatitis. The 188 patients with hepatitis consisted of 85 patients (45.2 %) without hepatic coma, and 103 patients with hepatic coma (54.8 %), with 54 patients (28.7 %) classified with the acute type and 49 patients (26.1 %) classified with the subacute type. The 23 patients without hepatitis were divided into 11 patients (47.8 %) without hepatic coma and 12 patients (52.2 %) with hepatic coma, including 7 (30.4 %) patients with the acute type and 5 (21.7 %) patients with the subacute type. In contrast, all patients with LOHF presented with hepatic coma and were classified as having the histological features of hepatitis (Fig. 1).

**Fig. 2:**
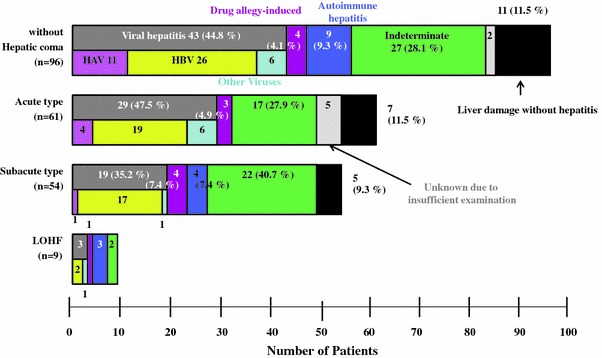
Etiology of acute liver failure in Japanese patients enrolled in a nationwide survey performed by the Intractable Hepato-Biliary Diseases Study Group in Japan [31]. *LOHF* late-onset hepatic failure, *HAV* hepatitis A virus, *HBV* hepatitis B virus

The etiologies of liver damage in patients with acute liver failure are shown in Fig. 2. Viral infection was determined as the cause in 43 of 96 patients (44.8 %) with acute liver failure without hepatic coma, 48 of 115 patients (41.7 %) with acute liver failure with hepatic coma, including 29 of 61 patients (47.5 %) with the acute type and 19 of 54 patients (35.2 %) with the subacute type, and 3 of 9 patients (33.3 %) with LOHF. The percentage of patients with liver injury caused by viral infections was smaller than that reported from the previous nationwide survey in Japan, especially in patients with acute type of liver failure with hepatic coma [1, 24–29]. In most of the cases of viral infection, the causative virus was HBV; transient infection was predominant in patients without hepatic coma and in those with acute-type liver failure with coma, whereas the incidence of asymptomatic carriers showing acute exacerbation of hepatitis was frequent in patients with subacute-type liver failure with coma. It is noteworthy that among the 25 asymptomatic carriers, there were 9 patients with de-novo HBV hepatitis, with negative test results for serum hepatitis B surface (HBs) antigen, who developed acute liver failure following therapy with immunosuppressive and/or anticancer drugs through an increase of the serum HBV-DNA level.

Although a small number of patients with autoimmune hepatitis and drug allergy-induced liver damage were found among patients with each disease type, the etiology of liver failure remained indeterminate in most of the remaining patients, including 27 of 96 patients (28.1 %) with acute liver failure without hepatic coma, 39 of 115 patients (33.9 %) with acute liver failure with coma (including 17 of 61 patients [27.9 %] with the acute type and 22 of 54 patients [40.7 %] with the subacute type of liver failure), and 2 of 7 patients (22.2 %) with LOHF. Etiologies other than hepatitis that may have induced liver injury were found in 11 patients (11.5 %) with acute liver failure without hepatic coma and in 12 patients (10.4 %) with acute liver failure with hepatic coma (including 7 patients [11.5 %] with the acute type and 5 patients [9.3 %] with the subacute type of acute liver failure), while hepatitis was the cause of the liver injury in all the patients with LOHF. The etiologies of liver damage other than hepatitis were circulatory disturbance in 6 patients, hepatic infiltration by malignant cells in 5 patients, postoperative liver injuries in 4 patients, metabolic disease in 3 patients, hemolytic-phagocytotic syndrome (HPS) in 3 patients, and drug toxicity-induced liver injury in 2 patients.

Among the 220 patients with acute liver failure and LOHF, 29 (13.2 %) underwent liver transplantation and the remaining 191 patients (86.8 %) were given conservative medical treatment, including artificial liver support (consisting of plasma exchange and hemodiafiltration). Liver transplantation was performed only in patients with hepatitis, while all of the patients without histological evidence of hepatitis received medical treatment alone. The survival rate of the 191 patients managed by medical treatment alone was 51.3 % (98/191), including 54.8 % (92/168) in patients with hepatitis and 26.1 % (6/23) in patients without hepatitis. In patients with hepatitis, the survival rate was 86.7 % (72/83) in the patients without hepatic coma, 31.7 % (13/41) in the patients with acute-type liver failure with coma, and 19.4 % (7/36) in the patients with subacute-type liver failure with coma, and 0 % (0/8) in those with LOHF; these values were greater than those in the patients without hepatitis:45.5 % (5/11) in the patients without hepatic coma, 14.3 % (1/7) in patients with acute-type liver failure with coma, and 0 % (0/5) in patients with subacute-type liver failure with coma. In contrast, the survival rate in the patients treated by liver transplantation was 62.1 % (18/29). The overall survival rate, including the patients treated by liver transplantation, was 52.7 % (116/220).

## Outcome prediction models and guideline for liver transplantation in patients with acute liver failure

Among the 220 patients with acute liver failure seen in 2010, 112 patients were diagnosed as having fulminant hepatitis or LOHF with the histological features of hepatitis [31]. The outcomes of these patients were as follows; 20 patients (17.9 %) and 65 patients (58.0 %), respectively, survived and died with medical treatment alone, and 27 patients (24.1 %) underwent liver transplantation. Liver transplantation is inevitable for the rescue of most patients with acute liver failure, even in Japan, where artificial liver support, including plasma exchange, is provided for almost all patients. Thus, outcome prediction models with high sensitivity and specificity levels were required to determine the indications for liver transplantation.

Liver transplantation has been recognized as the standard therapy for patients with acute liver failure in Europe and the United States since the 1980s [32, 33]. Bismuth et al. [34] reported that liver transplantation should be considered in patients with grade III or more severe hepatic encephalopathy, with plasma coagulation factor V activity levels of less than 20 % of the standardized values. Also, Emond et al. [35] reported that patients with brain edema due to encephalopathy, with prolongation of the prothrombin time after 24 or 48 h of intensive medical care, were candidates for liver transplantation. A similar outcome prediction model was published by Bernuau et al. [36] through multivariate analysis of the data of 115 patients with fulminant hepatitis due to HBV infection, in which plasma factor V activity levels, status of disappearance of HBs antigen, and the serum α-fetoprotein concentration were selected as independent predictors of survival. Moreover, in 1989, O’Grady et al. [37] published a guideline with selection criteria for liver transplantation in patients with acute liver failure, based on a multivariate analysis of the data of 588 patients seen between 1973 and 1985. In this guideline, the prognosis was estimated differently in patients with liver failure resulting from acetaminophen intoxication and in those with liver failure resulting from viral hepatitis or drug allergy-induced liver injury. In the former category of patients, the prognosis was estimated based on 3 parameters; namely, the arterial blood pH, peak prothrombin time, and the serum creatinine level. In contrast, in the latter category of patients, the prognosis was determined based on 5 parameters; namely, etiology of the disease, age of the patient, duration of jaundice before the onset of hepatic encephalopathy, peak prothrombin time, and the serum bilirubin level. This famous guideline, well known as the King’s College Hospital criteria, was widely used around the world to determine the indications for liver transplantation in patients with acute liver failure [38–40]. Also, the usefulness of the model for end-stage liver disease (MELD), which was originally established to evaluate the prognosis of chronic liver failure patients [41], was assessed in comparison with the predictive accuracy of the King’s College Hospital criteria in patients with acute liver failure [42–45].

However, the King’s College Hospital criteria were found to be of limited usefulness for patients with fulminant hepatitis in Japan [46], and the predictive accuracy of these criteria adopted for patients seen between 1993 and 1995 was found to be only 55 % for the assessment conducted at the onset of hepatic encephalopathy, and 53 % for the assessment conducted on day 5 after the onset of encephalopathy. Thus, a new guideline that could be adopted for patients in Japan was established by Sugihara et al., based on the results of a project undertaken by the Acute Liver Failure Study Group of Japan in 1996 [46]. According to this guideline, the prognosis of patients with fulminant hepatitis is estimated through a two-step procedure (Table 7). First, the estimated prognosis is determined at the onset of hepatic encephalopathy based on 5 parameters, with the parameters associated with a poor prognosis being: (1) age older than 45 years, (2) interval of 11 or more days from the onset of the initial disease symptoms to the development of grade II or more severe hepatic encephalopathy, (3) prothrombin time less than 10 % of the standardized value, (4) serum bilirubin level of 18 mg/dL or more, and (5) ratio of the serum direct to total bilirubin levels of less than 0.67. Patients fulfilling 2 or more of the above criteria, with the estimated prognosis of “death,” are enrolled as candidates for liver transplantation. Then, intensive therapy, including artificial liver support, is administered to these patients for 5 days if possible, and those showing improvement of both the prothrombin time and encephalopathy grade are excluded from the list of candidates for liver transplantation, with the estimated prognosis changed to “alive”. Such reassessment after intensive treatment for 5 days seemed to improve the prognostic accuracy of the guideline in fulminant hepatitis patients in Japan, where artificial liver support can be undertaken for more than 90 % of the patients [1, 24–29]. According to a prospective study, in which the guideline was adopted for patients seen between 1993 and 1995, the predictive accuracy of the guideline was 76 % for the first assessment and 82 % for the reassessment [46]. On the other hand, when the guideline was adopted for patients seen between 1998 and 2003, the predictive accuracy was even worse; the accuracy values in the patients not receiving liver transplantation were 67 and 78 % among those with acute and subacute types of fulminant hepatitis, respectively, and the specificity of the guideline was extremely low, especially in patients with the subacute type of fulminant hepatitis [47]. Thus, the guideline to determine the indications for liver transplantation in acute liver failure patients in Japan needs to be updated.

**Table 7:** Guideline to determine the indications for liver transplantation for patients with fulminant hepatitis and LOHF (published by the Acute Liver Failure Study Group of Japan in 1996); from references [46, 47]

Patients may be registered as potential recipients of liver transplantation when at least 2 of the following 5 criteria are satisfied at the time of the onset of grade II or more severe hepatic encephalopathy
1. Age ≥45 years
2. Interval from the appearance of the initial symptoms to the development of hepatic encephalopathy ≥11 days
3. Prothrombin time <10 % of the standardized value
4. Serum bilirubin concentration ≥18.0 mg/dL
5. Ratio of the direct to total bilirubin concentration <0.67
If liver transplantation cannot be performed within 5 days of the onset of hepatic encephalopathy and intensive medical therapy, including artificial liver support, is undertaken, the prognosis of the patients is evaluated again. If both of the criteria listed below are positive at 5 days after the onset of hepatic encephalopathy, the prognosis is reassessed as “alive” and the patients are excluded from the candidate list for liver transplantation
1. The hepatic encephalopathy shows improvement to grade I or less or attenuation by 2 or more grades
2. Prothrombin time improves to over 50 % of the standardized value

Accordingly, the task force of the Intractable Hepato-Biliary Diseases Study Group established a novel scoring system for predicting the outcomes of patients with fulminant hepatitis and LOHF in 2011, through analysis of the data of 1,096 patients enrolled in a nationwide survey [48]. In this system, 6 parameters were identified and graded as 0, 1, or 2; the parameters were: the interval between disease onset and the development of hepatic encephalopathy, prothrombin time, total serum bilirubin concentration, ratio of direct to total bilirubin concentration in the serum, peripheral blood platelet count, and presence/absence of liver atrophy (Table 8). The predicted mortality was greater than 90 % in patients with a total score of 7 or more, with predicted mortalities of 80–90, 70–80, and 50–60 % in those with a total score of 6, 5, and 4, respectively, while the predicted mortality was less than 30 % in those with a total score of 3 or less. When the prognosis of the patients with a total score of 5 or more was judged as “death”, the predictive accuracy was 0.80, with sensitivity, specificity, positive predictive value (PPV), and negative predictive value (NPV) of greater than 0.70 even in the validation cohort [48].

**Table 8:** Scoring system to predict the mortality of patients with fulminant hepatitis and LOHF established by the Intractable Hepato-Biliary Diseases Study Group in Japan in 2010; from reference [48]

Score	0	1	2
O-C (days)	≤5	6–10	11≤
PT (%)	20<	5<, ≤20	≤5
TB (mg/dL)	<10	10≤, <15	15≤
D/T ratio	0.7≤	0.5≤, <0.7	<0.5
PLT (10^4^/*μ*L)	10<	5<, ≤10	≤5
Liver atrophy	Absent	Present	

*PT* prothrombin time, *TB* total bilirubin, *D/T ratio* ratio of direct to total bilirubin concentration, *PLT* platelets, *O-C* the interval between hepatitis onset and hepatic encephalopathy development

Recently, we performed a cluster analysis of 1,022 patients with fulminant hepatitis and LOHF who were enrolled in a nationwide survey between 1998 and 2007, to evaluate the validity of the classification of acute liver failure in Japan; we used a self-organizing map (SOM), a data mining method that has been shown to be suitable for analyses of complex multidimensional relationships [49]. The results of the analysis revealed that the patients could be classified into three clusters, independent of the interval between the disease symptoms onset and the development of hepatic encephalopathy, with the clinical outcomes differing markedly among the clusters [49]. This observation prompted us to postulate that data-mining methods may be useful to revise the outcome prediction system, and we established a decision-tree algorithm for prediction of the prognosis of acute liver failure patients [50]. The outcome of the patients at the onset of encephalopathy was predicted based on 5 items: the patients were classified into 6 categories, with mortality rates ranging between 89 and 23 %. The outcome of the patients was also predicted based on 7 items at 5 days after the onset of encephalopathy; the patients were classified into 8 categories with mortality rates ranging between 100 and 11 %. Also, we established outcome prediction models based on other data-mining methods, such as radial basis function (RBF) and back propagation (BP) (unpublished data). The usefulness of these models based on data-mining methods needs to be further investigated.
